# Does the amygdala response correlate with the personality trait ‘harm avoidance’ while evaluating emotional stimuli explicitly?

**DOI:** 10.1186/1744-9081-10-18

**Published:** 2014-05-07

**Authors:** Peter Van Schuerbeek, Chris Baeken, Robert Luypaert, Rudi De Raedt, Johan De Mey

**Affiliations:** 1 Departement of Radiology, UZ-Brussel, Vrije Universiteit (VUB), Laarbeeklaan 101, 1090, Brussels, Belgium; 2 Departement of Psychiatry, UZ-Brussel, Vrije Universiteit Brussel (VUB), Brussel, Belgium; 3 Departement of Psychiatry and Medical Psychology, Ghent University, Ghent, Belgium; 4 Departement of Experimental, Clinical and Health Psychology, Ghent University, Ghent, Belgium

**Keywords:** fMRI, Harm avoidance, Affective personality, Anxiety-sensitivity, Amygdala, Amygdala subregions, Explicit processing, Emotion regulation

## Abstract

**Background:**

The affective personality trait ‘harm avoidance’ (HA) from Cloninger’s psychobiological personality model determines how an individual deals with emotional stimuli. Emotional stimuli are processed by a neural network that include the left and right amygdalae as important key nodes. Explicit, implicit and passive processing of affective stimuli are known to activate the amygdalae differently reflecting differences in attention, level of detailed analysis of the stimuli and the cognitive control needed to perform the required task. Previous studies revealed that implicit processing or passive viewing of affective stimuli, induce a left amygdala response that correlates with HA. In this new study we have tried to extend these findings to the situation in which the subjects were required to explicitly process emotional stimuli.

**Methods:**

A group of healthy female participants was asked to rate the valence of positive and negative stimuli while undergoing fMRI. Afterwards the neural responses of the participants to the positive and to the negative stimuli were separately correlated to their HA scores and compared between the low and high HA participants.

**Results:**

Both analyses revealed increased neural activity in the left laterobasal (LB) amygdala of the high HA participants while they were rating the positive and the negative stimuli.

**Conclusions:**

Our results indicate that the left amygdala response to explicit processing of affective stimuli does correlate with HA.

## Introduction

The heritable temperament trait ‘Harm Avoidance’ (HA) from the psychobiological model of personality [1-3] describes an individuals susceptibility to the feelings of fear and anxiety and his/her tendency to exhibit inhibition behavior [3]. The HA dimension ranges from neurotic introversion (high HA) to stable extraversion (low HA) [4]. It shows a strong positive correlation with neuroticism, a strong negative correlation with extraversion and a weak negative correlation with openness and conscientiousness [5], from the Big Five personality model. A high HA individual is characterized by an enhanced fear of uncertainty, by pessimism, extensive worries, shyness and proneness to fatigue. The HA trait has been demonstrated to be useful in the epidemiology and detection of depressions and anxiety disorders and to be predictive for their severity and treatment outcome [6-9]. Individuals prone to anxiety disorders or depressive states have been found to be more attentive to negative stimuli (attentional bias) and to rate positive and neutral stimuli as less positive (emotional bias) [10-13].

The left and right amygdalae are known to be key nodes in the processing of affective stimuli. Both amygdalae are subdivided into 3 subregions: the laterobasal (LB) amygdala mainly involved in determining the valence (positive or negative) and arousal (strength) of the observed emotion, the superficial (SF) amygdala mainly recruited in directing attention towards affective stimuli and finally the centromedial (CM) amygdala mainly involved in initiating behavioral responses [14-19]. The induced emotional responses are down regulated by cognitive processes in the prefrontal cortex by reappraisal of the stimuli and limiting the attention given to the stimuli [20-22].

The personality traits ‘trait anxiety’, ‘neuroticism’ [10,23-27] and HA [28] were found to correlate positively with the left and right amygdalae responses to fearful stimuli. These studies used a functional magnetic resonance imaging (fMRI) task in which the emotional stimuli were processed implicitly. More specifically, the volunteers were instructed to focus on the non-emotional stimuli presented following an emotional stimulus [28] or on a non-emotional feature in the presented facial expressions (e.g., color, age or gender) [10,24-26]. Ball et al. [27] asked their subjects to match faces by their facial expressions. These tasks mainly related individual differences in the attentional bias to amygdalae activation as that the presented emotional information was processed automatically and attracted the attention while it had to be ignored to perform the required task.

In a previous study [29] when trying to relate emotion induced amygdalae activity to the personality trait HA beyond the attentional modulation, the participants were instructed to simply observe attentively positive, negative and neutral stimuli without, performing any emotional or cognitive task. This study was based on an earlier study [30] in which differences in the lateralization of the amygdalae responses to affective stimuli were studied in low, average and high HA females. The volunteers were asked to focus on their emotions elicited while passively viewing the stimuli. This study revealed an increased left lateralized amygdala response to the negative stimuli in the high HA participants while no lateralization of the amygdalae response was observed in the low and average HA participants. Contrary to this, the [29] study revealed a negative correlation between the left amygdala activation and HA during the sustained processing of negative stimuli, probably due to an increased tendency in the high HA participants to shift attention away from the negative stimuli in an attempt to control the induced emotional reaction.

Compared to implicitly processed emotional stimuli, explicitly processed stimuli were found to evoke an increased response in the visual processing areas (the visual cortex, the fusiform gyrus and the associated temporal gyrus) due to the increased attentional load and a more detailed analysis of the stimuli and in the prefrontal cortex due to the increased cognitive control needed to perform the task [20,31-35]. As a result of these differences in visual processing and cognitive control during the explicit processing of affective stimuli, increased amygdalae responses were observed by some researchers [34] while others observed decreased amygdalae responses [31,33]. The amygdalae responses were found to be less active during an explicit valence rating task than in a passive viewing task [20,32] due to an increased top-down control from the prefrontal cortex.

An extension of the correlations between amygdalae activity and HA reported using implicit processing and passive viewing tasks could be hypothesized for explicit processing tasks. However, contrary to the attentional bias which has been consistently revealed in patients with affective disorders and is related to affective personality traits in healthy individuals, the emotional bias during facial recognition has only been reported in patients [36-38] but not in healthy individuals [37,39,40]. The observed bias in patients was found to be accompanied by an hyperactivation of both amygdalae while subjects rated negative expressions and an hypoactivation while they rated positive expressions.

In the current study, using fMRI we have tried to relate the activation of the different subregions of the amygdalae during the explicit processing of emotional stimuli to HA. We expected the activation in the LB amygdalae to increase with HA due to an increased sensory input from the visual processing areas while we did not expect to find such a correlation in the SF amygdalae and the CM amygdalae due to the increased cognitive control from the prefrontal and cingulate cortex.

## Materials and methods

### Participants

To exclude effects from gender, age and disease state [41,42], the study cohort was restricted to healthy young female Belgian natives (34 volunteers, age range: 19–27 years) recruited by local advertising among staff members and students at our hospital and the participating universities: Vrije Universiteit Brussel (VUB) and Ghent University. All volunteers were Caucasian and two participants were mothers. Each participant was required to be medication-free (except for birth control medication), right-handed (as assessed with the Van Strien questionnaire [43]), free of any anxiety or depressive disorder (as assessed with the Dutch version of the Mini-International Neuropsychiatric Interview (Mini) [44]), without any personal psychiatric disorder history and non-depressed (defined as having a score lower than 9 on the 21 item Beck Depression Inventory (BDI-II) [45]). All volunteers gave their written informed consent and were financially compensated. The study was approved by the Institutional Ethical Board of the University Hospital of the Vrije Universiteit Brussel (UZ Brussel) and in accordance with the guidelines laid down in the declaration of Helsinki [46].

### TCI questionnaire

All participants completed the Dutch version of the Temperament and Character Inventory (TCI) questionnaire [47] by answering “True” or “False” to 240 statements. Based on this questionnaire a HA score on a scale from 0 to 40 was determined for each participant.

### MRI imaging

All scans were performed on a 1.5 T Philips Intera MRI system (Philips, Best, The Netherlands) with a six-channel SENSE head coil. For anatomical reference, a 3D T1-TFE MRI scan (TI/TR/TE = 1501/16/4.6 ms, flip angle = 30°, FOV = 240 × 240 × 200 mm, resolution = 1 × 1 × 2 mm and 100 axial slices) was measured. The fMRI scans were obtained using an FFE-EPI sequence (TR/TE = 3000/35 ms, flip angle = 90°, FOV = 240×240 mm, resolution = 3.75×3.75 mm, slice thickness/gap = 5.0/1.0 mm, 18 slices) with 2 dummy scans and 168 dynamics.

### fMRI paradigm

**Figure 1:**
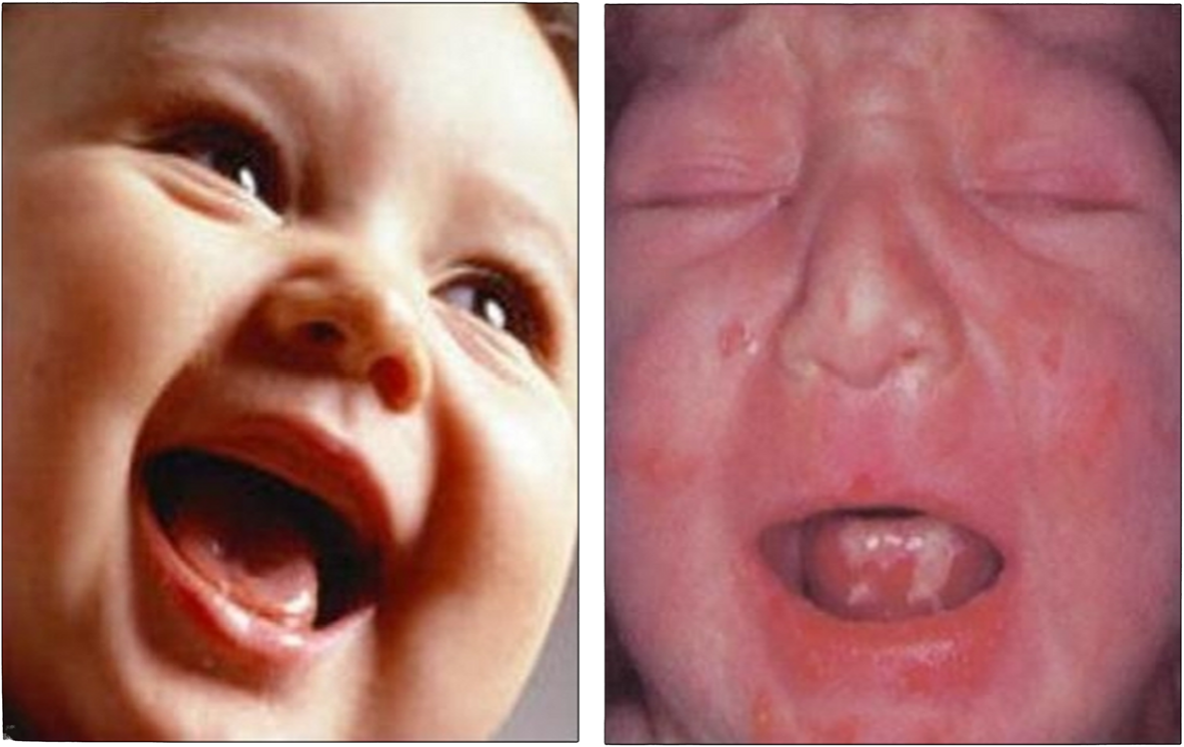
Example of a positive (left) and a negative (right) stimulus.

The emotional stimuli consisted of a set of 26 pictures of smiling baby faces (positive stimuli) and 25 pictures showing crying baby faces with severe dermatological ailments (negative stimuli). These pictures were similar to the stimuli used in our previous studies [29,30]. The pictures for the positive stimuli were collected from family photos from staff members and from the Internet, while those for the negative stimuli originated from the dermatological literature. All babies were Caucasian and their estimated mean age was 5.5 months (SD = 4.0 months). All pictures showed a single male or female baby face (depicting only the facial expressions with the eyes, nose and mouth) directly looking at the camera (Figure 1). All were rendered at the same resolution (275×360), matched for color and luminosity and presented on a white background. Each picture was used 2 to 3 times to yield a total of 68 positive and 68 negative stimuli.

The choice of stimuli was motivated by the fact that earlier reports had shown baby faces to engage attention in young females and to induce spontaneous emotional reactions [30,48,49]. The reason for selecting crying baby faces with a severe dermatological condition for the negative stimuli, was to avoid emotional ambiguity and to make sure they elicited an unequivocally negative, aversive reaction rather than sympathy and the desire to console. The subjects were familiar with the stimuli, as they had also participated in an earlier fMRI study [30] using a different, but similar set of stimuli.

Valence and arousal ratings for all pictures were collected in an independent but similar group of females prior to this study. The negative stimuli were found to have a mean valence score of 1.50 (SD = 0.34) and a mean arousal score of 7.79 (SD = 0.49). The positive stimuli were found to have a mean valence score of 7.02 (SD = 0.47) and a mean arousal score of 5.65 (SD = 0.49). Independent samples T-tests revealed a significant difference in valence (positive versus negative: t(49) = 47.49, *p <* 0.01) and a significant difference in arousal (positive versus negative: t(48) = -15.41, *p* < 0.01).

The pictures were projected through the window of the MRI room onto the back of a tracing-paper screen. This screen, placed at 2 m from the magnet center, was observed by the volunteers via a mirror fixed on top of the head coil. The Presentation software [50] was used for presenting the stimuli, separated by a fixation-cross picture in a randomized order following a jittered inter-stimulus timing (range 2026–13186 ms) for a duration of 1000 ms. The optimal timing and order of the stimuli were determined in advance using the Matlab toolbox ‘OptimizeDesign’ [51]. The participants were instructed to rate the valence of the facial expressions as fast as possible by pressing buttons on a pair of MRI compatible response boxes (Current Designs, Philadelphia, USA) by their left (negative) or right (positive) thumb.

### Analysis

Preprocessing and analysis of the fMRI data were performed in SPM8 (Statistical Parametric Mapping, Wellcome Department of Imaging Neuroscience, London, UK) running in Matlab (R2010a).

#### Preprocessing and processing of the individual scans

The fMRI volumes were realigned to the first volume to correct for residual motion, slice-time corrected to correct for time shifts between the measurement of consecutive slices, normalized to the EPI MNI template (Montreal Neurologic Institute) and smoothed with an isotropic 8 mm FWHM Gaussian filter. The 3D anatomical images were normalized to the T1 MNI template.

For each volunteer a design matrix with eleven regressors was constructed on the basis of the timings of the picture presentations for each emotional condition, convolved with the canonical hemodynamic response function (HRF) and its time derivative, six motion regressors (3 translation, 3 rotation) to take residual motion into account and a constant to model the activation onset. This model was fitted to the measured data using the generalized linear model (GLM) approach.

As we expected to encounter brain areas processing the emotional stimuli both dependent and independent of the stimulus valence, the response to the positive and the negative stimuli was calculated separately. These responses were derived from the fitted parameters (betas) as the magnitude of the HRF based on [52] and using the Matlab scripts from [53]. The baseline with respect to which these responses were calculated was the mean of all activity going on locally during the experiment and not explained by the model. This approach is similar to measuring the mean neural activity in identical experimental conditions but with only a fixation-cross present and omitting the emotional stimuli. The resulting neural responses to the positive and the negative stimuli were correlated separately to trait HA (see section 2.5.3).

We did not study the difference between the response to the positive stimuli and the response to the negative stimuli as is regularly done in emotional fMRI studies. This approach was motivated by the fact that subtracting the positive and the negative neural responses (contrast ‘positive – negative’ or ‘negative - positive’) cancels common neural activity related to the perception and the basic analysis of the stimuli that is independent of the stimulus valence. This common activity could be of interest since the amygdalae are known to be activated by positive, negative and neutral facial stimuli [15]. If the amygdalae responses to the positive stimuli and to the negative stimuli would exhibit a similar dependence on HA, the subtraction of positive and negative neural activations would not depend on HA as this difference would be constant. To test for differences in amygdalae responses between the low and high HA participants dependent and independent of the stimulus valence, an analysis of variance (ANOVA) with group and valence as factors was performed in addition to the correlation analyses (see section 2.5.4).

#### Significance tests for the responses to the stimuli

To test whether our paradigm succeeded in generating a significant response in all brain areas involved in facial recognition, generating an emotional response and cognitive control, we performed separate 1-sample T-tests for the response to the positive and to the negative stimuli. In these analyses HA was not taken into account.

#### Correlation between the neural responses and HA

To study the correlation between HA and the neural responses to the positive and the negative stimuli, we performed regression analyses based on the individual response maps. In these regression analyses the HA scores were used as covariate of interest. A constant was included in the regression to model the mean neural response.

#### ANOVA comparing the low and high HA participants

Since correlation analyses have the inherent drawback that they only test for a linear relationship between neural activity and HA, a 2×2 ANOVA was performed to test for differences in neural activity between the low and high HA participants without this linear assumption. For this analysis the subject sample was subdivided into low and high HA subgroups based on the median HA score. Group was used as a between-subjects factor and valence as a within-subjects factor. To test for differences between both groups independent of the stimulus valence, the main effect of group was determined. To test for differences between both groups related to the stimulus valence, the interaction effect ‘group × valence’ was determined. We did not investigate for the main effect of valence since that is similar to the contrast ‘positive versus negative’ averaged over the whole subject group.

#### Whole brain analyses restricted to the amygdala

In order to focus on the left and right amygdalae we defined a mask in the WFU-Pickatlas toolbox [54-56] masking the whole brain except for the amygdalae as defined in the AAL atlas. Using this mask, we repeated the 1-sample T-tests, the regression analyses and the ANOVA. We used the probabilistic cytoarchitectonic maps of [57], as freely available in the SPM anatomy toolbox v1.7 [58], to assign the results to the corresponding amygdalae subregions.

#### Correction for multiple comparisons

A general problem in neuroimaging studies is the risk for type I and II errors, since each statistical test is performed on each unmasked image voxel separately. To take care of this problem, a multiple-comparison correction was performed. As the classical Bonferroni correction is known to be too conservative for use in fMRI studies, we applied a cluster-extent threshold in addition to the voxel significance threshold (*p* ≤ 0.005 (1-tailed)), taking into account that the chance of finding a whole cluster by chance drops when the cluster size increases [59]. To determine this cluster-extent threshold, we performed 1000 Monte Carlo simulations using AlphaSim [60,61] to obtain a final corrected significance *p* ≤ 0.05 (1-tailed). As the result of these simulations depends on the average correlations between neighboring voxels derived from the statistical map given as input to AlphaSim, we performed these simulations separately for each statistical test. Since all analyses were performed twice (once with a mask masking the background and leaving the whole brain unmasked and once with all image voxels masked except for those in the left and right amygdalae) and the cluster-extent threshold depends on the mask, the simulations were also performed twice.

## Results

### Personality assessment

The measured TCI scores fell in a range of 2–25 for HA with 13 as median score. To perform the ANOVA, the subjects were subdivided into a low HA group (16 participants) having a HA score less than the median and a high HA group (17 participants) with a HA score equal to or above the median.

### Behavioral results

Due to a technical problem, only 26 response files could be recovered and used for the behavioral analyses. The mean response time for the positive stimuli was 633 ms (SD = 79 ms) while the mean response time for the negative stimuli was 691 ms (SD = 129 ms). A maximum of 12 stimuli were rated erroneously. Maximal 8 positive stimuli have been rated as negative and 10 negative stimuli as positive. Paired T-tests revealed a significantly increased response time for the negative compared to the positive stimuli (t(25) = 3.03, *p* = 0.01) but failed to reveal a significant difference between the number of negative stimuli rated as positive and the number of positive stimuli rated as negative (t(25) = 0.61, *p* = 0.55).

Correlation analyses for response times, the number of misjudged valences, age and HA revealed a significant correlation between the response time for the positive stimuli and the response time for the negative stimuli (R = 0.65, *p* < 0.01), between the response time for the positive stimuli and age (R = -0.42, *p* = 0.03), between the response time for the negative stimuli and the number of negative stimuli rated as positive (R = 0.46, *p* = 0.02), between the response time for the negative stimuli and age (R = -0.61, *p* < 0.01), between the number of positive stimuli rated as negative and age (R = -0.43, *p* = 0.03) and between the total number of misjudged stimulus valences and age (R = -0.50, *p* = 0.01). No significant correlations were found between the response times and HA (positive: R = -0.13, *p* = 0.52; negative: R = -0.23, *p* = 0.25) nor between the number of valence misjudgments and HA (positive: R = 0.24, *p* = 0.25; negative: R = -0.08, *p* = 0.69; total: R = 0.07, *p* = 0.72). None of these correlations survived Bonferroni correction for multiple comparisons.

Fourteen of the recovered files corresponded to participants from the low HA group and 12 to participants from the high HA group. For the low HA participants, the mean response time for the positive stimuli was 645 ms (SD = 71 ms) while the mean response time for the negative stimuli was 680 ms (SD = 148 ms). For the high HA participants, these same response times were 654 ms (SD = 86 ms) and 705 ms (SD = 107 ms) respectively. The low HA participants rated on average 1 (SD = 1) positive stimulus as negative and 2 (SD = 2) negative stimuli as positive, while the high HA participants rated on average 3 (SD = 2) positive stimuli as negative and 2 (SD = 3) negative stimuli as positive. The 2-sample T-tests performed on the behavioral data failed to reveal a significant difference between the low and high HA group for the response time for the positive stimuli (*p* = 0.21), the response time for the negative stimuli (*p* = 0.22), the number of positive stimuli rated as negative (*p* = 0.08), the number of negative stimuli rated as positive (*p* = 0.86) and the total number of misjudgments of the valences (*p* = 0.26). As none of these results were significant, no Bonferroni correction was applied.

All behavioral analyses were conducted in PSPP 0.7.9 [62].

### Image analyses

Due to the limited spatial resolution of the fMRI images, large clusters spanning several brain areas resulted from the analyses. For each cluster all brain areas covered were reported. The anatomical labels were determined using the Automatic Anatomical Labeling toolbox (AAL) [56].

#### Significant responses to the positive stimuli

The Monte Carlo simulations using the background-only mask delivered a cluster-extent threshold of 767 voxels for the response to the positive stimuli. This threshold applied in combination with a voxel significance threshold *p* < 0.005 revealed an activation of the neural response in the left and right visual cortex, the left sensorimotor cortex, the right ventrolateral prefrontal cortex (VLPFC) and in the left limbic cortex. A deactivation was observed in the left and right association cortex, the ventral visual processing system, the sensorimotor cortex, the medial frontal cortex and the right temporal cortex. A more detailed summary of the results of this analysis can be found in Additional file 1.

The Monte Carlo simulations using the brain mask masking the whole image except for the amygdalae, revealed a minimum cluster-extent threshold of 3 voxels. This threshold applied in combination with a voxel significance threshold *p* < 0.005 did not reveal any activation or deactivation in the left or right amygdala.

#### Significant responses to the negative stimuli

The Monte Carlo simulations using the background mask, delivered a cluster-extent threshold of 762 voxels for the response to the negative stimuli. This threshold applied in combination with a voxel significance threshold *p* < 0.005 revealed an activation in a large cluster covering the visual cortex, the sensorimotor cortex, the prefrontal cortex and the limbic cortex and in a cluster located in the left prefrontal cortex. The brain deactivated in response to the negative stimuli in the left association cortex, the sensorimotor cortex, the ventral visual processing system, the left visual eye field, the right association cortex and in the medial frontal cortex. A more detailed summary of the results can be found in Additional file 2.

**Figure 2:**
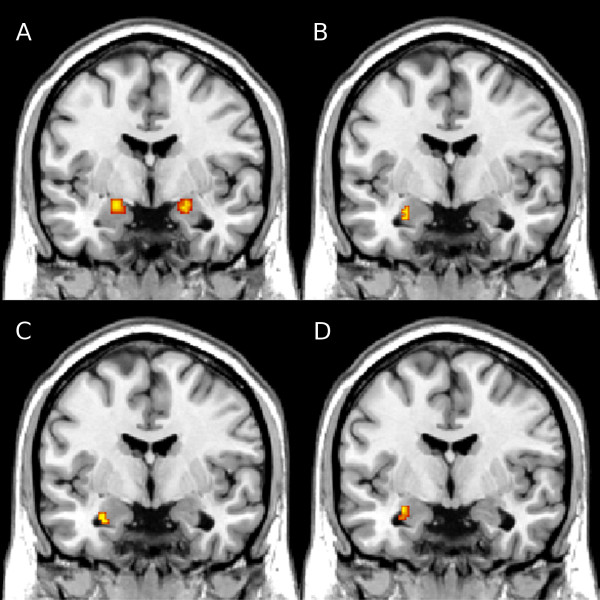
**The cluster results observed in the amygdalae, overlaid on an anatomical template.** The figures present the observed responses to the negative stimuli **(A)**, the observed correlation between the response to the negative stimuli and HA **(B)** and between the response to the positive stimuli and HA **(C)** as well as the observed main effect of group **(D)**.

The Monte Carlo simulations using the mask masking everything except the amygdalae, led to a minimum cluster-extent threshold of only 1 voxel. This threshold applied in combination with a voxel significance threshold *p* < 0.005 uncovered an activation in the left amygdala (cluster size = 36 voxels, mean t(32) = 3.42 (SD = 0.40), cluster peak at (-20,-4,-16)) and in the right amygdala (cluster size = 36 voxels, mean t(32) = 3.22 (SD = 0.34), cluster peak at (20,-4,-16)). The anatomy toolbox revealed that 85.4% of the activation observed in the left amygdala was located in the SF amygdala and 9.7% in the LB amygdala. The activation observed in the right amygdala, was located for 76.0% in the SF amygdala and for 1.0% in the LB amygdala. Figure 2A presents the observed activation clusters overlaid on an anatomical template.

#### Correlations between the neural response to the positive stimuli and HA

The Monte Carlo simulations with only the background masked, produced a cluster-extent threshold of 492 voxels for the regression analysis between the response to the positive stimuli and HA. Applying this threshold in combination with a voxel significance threshold *p* < 0.005 revealed a positive correlation in the left and right orbitofrontal cortex (OFC) and dorsolateral prefrontal cortex (DLPFC) and in the right visual cortex but failed to show any negative correlation. A more detailed summary of these results is presented in Table 1.

**Table 1:** Correlations between the neural response to the positive stimuli and HA

**Correlations between the neural response to the positive stimuli and HA**			
**Positive correlation**			
**Cluster size (voxels)**	**Position cluster peak (mm)**	**Mean t (SD)**	**Anatomical labels**
2841	(28,42,30)	3.38 (0.60)	Right middle and superior frontal cortex
			Right triangular and opercular inferior frontal gyri
			Right medial superior frontal cortex
			Right inferior, middle and superior orbitofrontal cortex
			Right medial orbitofrontal cortex
			Right caudate nucleus
			Right putamen
			Right insular cortex
1026	(-22,64,10)	3.29 (0.51)	Left middle and superior frontal cortex
			Left triangular inferior frontal gyrus
			Left medial superior frontal cortex
			Left inferior, middle and superior OFC
			Left medial OFC
552	(32,-74,-6)	2.32 (0.46)	Right middle and superior occipital cortex
			Right calcarine gyrus
			Right fusiform gyrus

These results were found after applying a voxel significance threshold *p* < 0.005 and a cluster-extent threshold Ke > 492 voxels.

**Figure 3:**
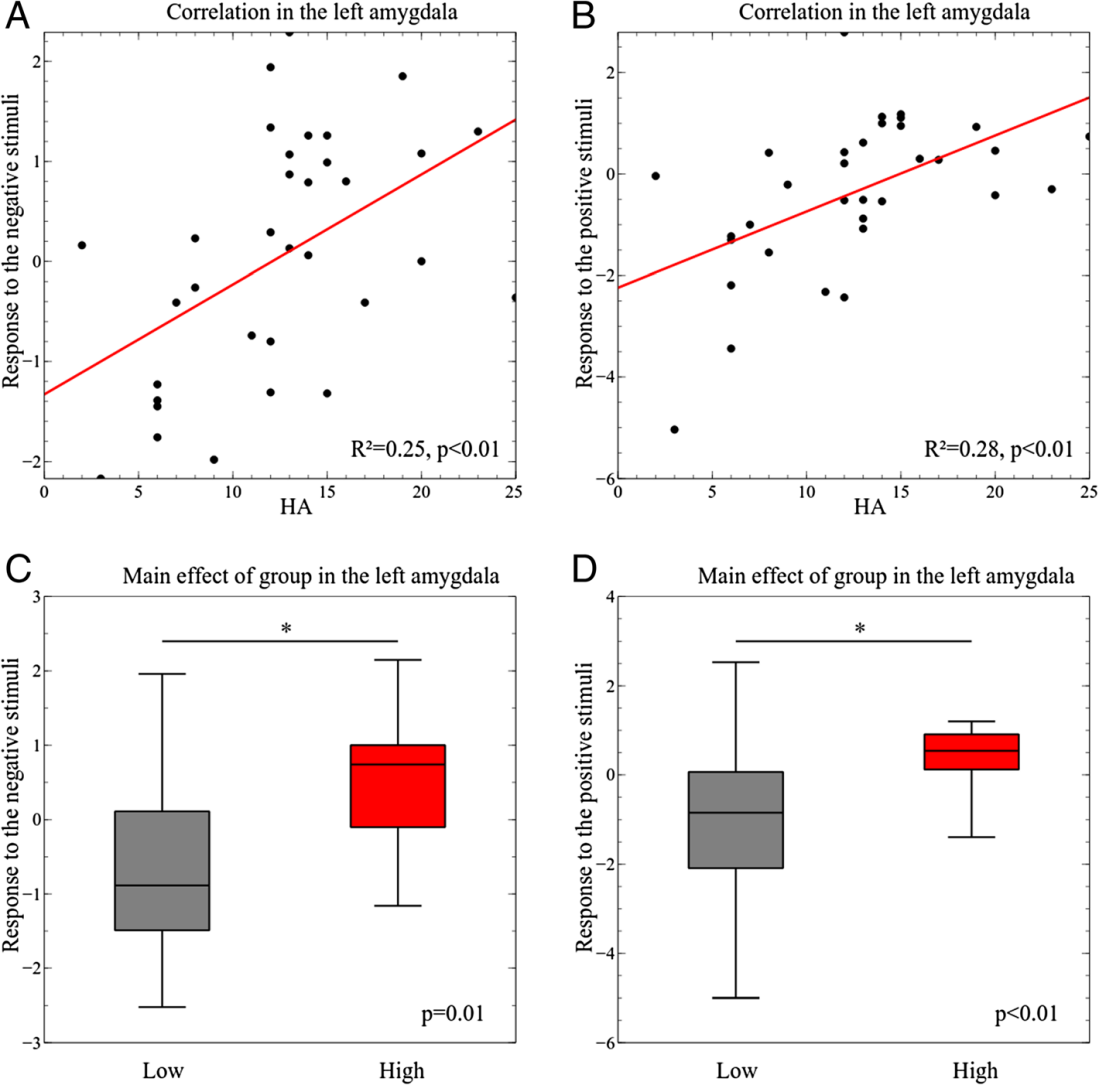
**Correlation and box plots presenting the observed correlations and main effect in the left amygdala.** The plots show the observed correlation between the left amygdala response to the negative stimuli and HA **(A)** and between the left amygdala response to the positive stimuli and HA **(B)**. The box plots exhibit the left amygdala response to the negative stimuli **(C)** and the response to the positive stimuli **(D)** in the low and high HA group. The whisker bars from the box plots presents the minimal and maximal neural response measured. The asterisks indicates significant group differences.

The Monte Carlo simulations using the brain mask masking everything except the amygdalae, produced a minimum cluster-extent threshold of only 1 voxel. Applying this threshold in combination with a voxel significance threshold *p* < 0.005 revealed a positive correlation between the neural response to the positive stimuli and HA in the left amygdala (cluster size = 9 voxels; mean t(31) = 3.22 (SD = 0.33); cluster peak at (-28,-4,-24)). No correlation between the right amygdala response to the positive stimuli and HA was found. Using the probabilistic cytoarchitectonic maps, 100.0% of the correlation observed in the left amygdala was assigned to the LB amygdala. Figure 2B presents the observed correlation cluster overlaid on an anatomical template. The right plot in Figure 3 presents the observed correlation.

#### Correlations between the neural response to the negative stimuli and HA

The Monte Carlo simulations using the background mask, produced a cluster-extent threshold of 205 voxels for the regression analysis between the response to the negative stimuli and HA. The application of this threshold in combination with a cluster significance threshold *p* < 0.005 only revealed a positive correlation in the left middle cingulate cortex (MCC) (cluster size = 247 voxels; mean t(31) = 3.24 (SD = 0.38)).

The Monte Carlo simulations masking everything except the amygdalae, led to a minimum cluster-extent threshold of only 1 voxel. This threshold in combination with a voxel significance *p* < 0.005 revealed a positive correlation between the neural response to the negative stimuli and HA in the left amygdala (cluster size = 11 voxels; mean t(31) = 3.02 (SD = 0.17); cluster peak at (-28,-6,-18)). In the right amygdala, no correlations between the neural response to the negative stimuli and HA were observed. Using the probabilistic cytoarchitectonic maps, 83.0% of the cluster observed in the left amygdala was assigned to the LB amygdala and 17.0% to the SF amygdala. Figure 2C presents the observed correlation cluster overlaid on an anatomical template. The left plot in Figure 3 presents the observed correlation.

#### ANOVA: main effect of group

The Monte Carlo simulations using the background mask, resulted in a cluster-extent threshold of 58 voxels for the main effect of group. Using a maximal voxel significance of 0.005 significant main effects of group were observed in the left and right frontal cortex, the middle cingulate cortex and in the right visual cortex. In all these regions, post-hoc tests revealed a higher neural activity in response to the positive and the negative stimuli in the high HA participants than in the low HA subjects. A more detailed summary of these results is presented in Table 2.

**Table 2:** Main effects of group presenting activation differences independent of the stimulus valence

**ANOVA analysis: main effect of group**					
**Cluster size (voxels)**	**Position cluster peak (mm)**	**Peak F**	**Anatomical labels**	**Post-hoc tests**	
				**P: High – Low mean t (SD)**	**N: High – Low mean t (SD)**
135	(-32,22,36)	23.03	Left middle frontal cortex	3.01 (0.41)	2.82 (0.40)
			Left precentral gyrus		
186	(24,48,30)	23.00	Right middle and superior frontal corte	3.18 (0.34)	2.86 (0.57)
98	(22,22,4)	18.36	Right caudate nucleus	3.38 (0.44)	2.44 (0.47)
			Right putamen		
157	(42,50,2)	17.83	Right middle frontal cortex	3.07 (0.28)	2.68 (0.31)
			Right orbital middle and ingerior frontal cortex		
141	(-4,-14,32)	17.09	Bilateral middle cingulate cortex	3.51 (0.41)	2.40 (0.39)
118	(32,-74,14)	16.48	Right middle and superior occipital cortex	3.54 (0.33)	2.04 (0.26)

These results were found after applying a voxel significance threshold *p* < 0.005 and a cluster-extent threshold Ke > 58 voxels.

The Monte Carlo simulations masking everything except the amygdalae, yielded a minimum cluster-extent threshold of only 1 voxel. Applying this threshold in combination with a voxel significance threshold *p* < 0.005 only produced a main effect of group in the left amygdala (cluster size = 10 voxels, peak F(1,62) = 16.69, cluster peak at (-28,-6,-18)). Post-hoc tests revealed a higher neural activity in this cluster in response to the positive and the negative stimuli in the high HA participants. Based on the probabilistic cytoarchitectonic maps, 97.5% of this cluster was assigned to the LB amygdala and 2.5% to the SF amygdala.

#### ANOVA: interaction ‘group x valence’

The Monte Carlo simulations using the background mask, led to a cluster-extent threshold of 39 voxels for the interaction effect of group and valence. Significant interaction effects were observed in the left medial frontal cortex and the anterior cingulate cortex (ACC), in the left middle frontal cortex, the left orbitofrontal cortex (OFC) and in the right precentral and postcentral gyri. Post-hoc tests revealed a higher neural activity in the high HA participants in response to the positive stimuli but a lower neural activity in response to the negative stimuli in all these regions. A more detailed summary of these results is presented in Table 3.

**Table 3:** Interaction effects ‘group x valence’ presenting activation differences dependent on the stimulus valence

**ANOVA analysis: interaction ‘group × valence’**					
**Cluster size (voxels)**	**Position cluster peak (mm)**	**Peak F**	**Anatomical labels**	**Post-hoc tests**	
				**P: High – Low mean t (SD)**	**N: High – Low mean t (SD)**
118	(-8,26,36)	19.56	Left medial superior frontal cortex	2.06 (0.42)	-1.15 (0.50)
			Left anterior and middle cingulate cortex		
47	(-22,38,10)	19.35	Left middle frontal cortex	1.64 (0.49)	-1.49 **(**0.59**)**
61	(-34,48,-2)	16.28	Left orbital middle frontal cortex	2.73 (0.26)	-0.41 (0.28)
			Left middle frontal cortex		
41	(38,-22,42)	13.92	Right postcentral gyrus	1.62 (0.34)	-1.44 (0.31)
			Right precentral gyrus		

These results were found after applying a voxel significance threshold *p* < 0.005 and a cluster-extent threshold Ke > 58 voxels.

The Monte Carlo simulations masking everything except the amygdalae, yielded a minimum cluster-extent threshold of only 1 voxel. However, no interaction effects were observed in the amygdalae.

### The amygdala responses related to the behavioral results and the personality traits

For completeness we also analyzed the correlation between the amygdalae responses to the positive and negative stimuli and age, the measured response times and the number of misjudgments of the valences. These analyses revealed a negative correlation between the left amygdala response to the positive stimuli and the number of negative stimuli rated as positive (cluster size = 2 voxels; mean t(25) = 3.03 (SD = 0.04); cluster peak at (-16,-4,-16)) assigned to the SF amygdala (100%).

## Discussion

In this study, we hypothesized individual differences, dependent on the participants HA scores in the amygdalae activations observed during the explicit evaluation of emotional stimuli. In the high HA participants, the correlation analyses and the ANOVA revealed an enhanced response to the positive and to the negative stimuli in the left LB amygdala. The LB amygdala is known to be involved in processing of the sensory input coming from the visual cortex and the fusiform gyrus [19]. In high HA individuals, the increased visual input was hypothesized to result from an enhanced attentional bias. It has been shown that an enhanced attention towards facial expressions increases the response in the neural system responsible for the perception and the analysis of stimuli [63-65]. More specifically, an increased attentional load will boost the neural activity in the occipital cortex, the temporal cortex and the fusiform gyrus. In line with the hypothesized attentional bias, our whole-brain correlation analyses revealed a positive correlation between the response to the positive stimuli in the visual cortex and the fusiform gyrus and HA. Moreover, a significantly higher neural activation was observed in the high compared to the low HA participants in the right visual cortex independently of the stimulus valence. These observed differences are in agreement with the results of [66], who reported enhanced visual processing in anxious individuals related to their increased attentional bias as revealed by their eye-tracking results. Supplementary to our results [67], reported individual differences, dependent on the subjects HA scores, in the LB amygdalae connectivity with the visual cortex and fusiform gyrus. These differences in connectivity were most clearly observed in their female subjects.

The ANOVA and correlation analyses failed to reveal any activation difference in the CM and SF amygdalae between the low and high HA participants. The CM amygdalae are involved in the generation of the emotional output while the SF amygdalae are an intermediate station between the input from the visual processing areas and the prefrontal cortex [19]. Both subregions have connections with the prefrontal cortex which were found to correlate with HA [67]. However, these correlations were mainly observed in males. Through these connections, the prefrontal cortex is able to inhibit the neural activity in the CM amygdalae. Current theories hypothesize that emotion regulation and the inhibition of the amygdalae responses to affective stimuli are initiated in the VLPFC and continues over a neural network including the DLPFC, the MCC, the ACC, the insular cortex and the superior temporal cortex [68]. It has been shown that explicitly processing affective stimuli requires an increased cognitive control of the induced emotional responses from these areas [20,31-35]. Our whole brain results revealed that the neural activity in the prefrontal cortex and the cingulate gyrus differs between the low and high HA participants. In general, the neural activity in the prefrontal cortex was found to be higher in the high HA participants. These findings indicate that these participants had to make more efforts to regulate the induced emotional responses during explicit processing of the affective stimuli. We hypothesized that these increased efforts provided an explanation for the absence of any difference in the behavioral responses between the low and high HA participants. The behavioral results were in line with the behavioral results reported in [40]. In agreement with our whole-brain findings [69], reported increased neural correlates of the inhibition of negative emotional information in subjects at family risk to develop a major depressive disorder.

In clinical populations with an anxiety or depressive disorder, response differences while evaluating emotional stimuli were reported in the insular cortex, ACC, MCC, VLPFC and DLPFC in addition to increased amygdalae activations [70,71]. Unfortunately, these studies did not assign their findings to the amygdalae subregions. These results suggest that depressive patients or patients with an anxiety disorder do not only have impairments in their emotional responses but also in the cognitive and attentional regulation of these induced responses. Functional connectivity studies revealed that impairments in the down-regulation of the amygdalae responses from the prefrontal cortex could be causal for affective disorders [72-74]. Interestingly [72], was able to show this in females with a major depressive disorder while they where processing negative stimuli as well as positive stimuli. As we excluded subjects with symptoms of an affective disorder or a BDI above 9, it was not possible to relate our findings to clinical symptoms.

The results of the current study are in line with our previous papers [29,30]. In these papers we reported left lateralized amygdala responses to negative stimuli in high HA females [30] and a correlation between the left amygdala activation while passively watching negative stimuli and HA [29]. Others also reported differences between anxious and non-anxious individuals in their left amygdala responses to negative stimuli [27] or correlations between bilateral amygdalae responses to negative stimuli and HA [28] or anxiety [25]. Etkin et al. [10] revealed a positive correlation between the right amygdala activity and trait anxiety. Unfortunately, these previous studies did not assign their findings to the amygdalae subregions. Although it has been shown that the amygdalae respond to positive stimuli as well [75], most of these studies did not include positive stimuli in their paradigm. The current study seems to indicate similar differences related to trait HA in amygdalae responses in response to positive stimuli as to negative stimuli.

In general, these findings imply that individual differences in the neural responses induced by affective stimuli can be partly explained by individual differences in the personality trait HA. The current study extends these findings to the situation in which the participants had to evaluate the affective stimuli explicitly. While during implicit processing or passive viewing of affective stimuli an enhanced emotional response and attentional control has been observed [28,29], our new results point at differences in the cognitive control needed to perform the explicit task. In healthy females, this enhanced cognitive processing seems to be sufficient to inhibit increased emotional response. Unfortunately, our subject group did not include healthy females with a very high HA score (above 25). This limited range of HA scores limits the interpretation of our results to low, moderate and high HA females and the findings cannot be extended to healthy females with a very high HA score.

Although lateralization studies revealed that both amygdalae respond to emotional stimuli, the left and right amygdalae are found to be involved in different ways in emotional processing. The left amygdala is hypothesized to be involved in processing the emotional valence and arousal of the stimuli while the right amygdala is involved in the fast detection of emotional content in a stimulus [76,77]. Given that only the left amygdala response correlated with HA in the current and previous studies [29,30], our findings seem to indicate that only the processing of the valence and arousal of the affective stimuli is related to HA but not the fast detection of these stimuli.

Remarkably, although the neural response in the left LB amygdala correlated significantly with HA, the underlying response was found to be non-significant. As presented in the plots in Figure 3, an explanation for this is that the left LB amygdala deactivated in the low HA participants while it activated in the high HA participants. The performed T-test evaluated whether the mean response from all participants, independent of their HA score, was significantly different from 0.

The only significant amygdalae response observed was an activation of the left and right SF amygdalae, induced by the negative stimuli. This response did not correlate with HA or differ between the low and high HA participants. The SF amygdala is known to be sub-specialized into directing the attention towards socially relevant stimuli and processing the basic emotion of disgust [19]. We hypothesized these observed responses to be indicative for an increased feeling of disgust induced by the negative stimuli due to the dermatological ailments present in the baby faces, in all participants independent of their HA score. These increased feelings of disgust resulted in an increased response time after viewing a negative stimulus. In line with this interpretation, in [29] we had already reported a significant increase in the feelings of disgust (t(19) = 5.69, *p* < 0.01) in a similar group of healthy females after viewing the negative stimuli outside the MRI environment.

Some final remark should be made regarding the assignment of the findings to the amygdalae subregions. In the current study, this assignment was done using the probabilistic cytoarchitectonic maps of [57]. Although, these probabilistic maps were specifically designed for use in fMRI studies, some caution should exercised regarding the obtained results. Due to the limited spatial resolution of fMRI images, the performed smoothing step and the correlations between neighboring voxels, the accuracy of the assignment of the clusters observed in the amygydalae to the small subregions is rather limited. However, it would be of interest for studies of the relations between amygdalae activations and personality traits or affective disease states to assign their results to the amygdalae subregions given their different roles in affective processing.

## Limitations of the study

A major limitation of the current study is the limited sample size. The sample size was similar to that used by others in similar studies (e.g., [10]: 17 participants, [27]: 45 participants, [28]: 29 participants and [26]: 20 participants). This limited sample size could have resulted in a lack of power, increasing the chance of reporting false negative results (type II errors) aand false positive results (type I errors) [78]. To make an acceptable balance between the chances for reporting type I and type II errors, we performed Monte Carlo simulations in AlphaSim [60,61] for each analysis. These simulations revealed rather conservative cluster-extent thresholds in combination with the voxel significance threshold of *p* ≤ 0.005 selected to look at the whole brain results.

The setup of this study implies some limitations on the scope of our results and conclusions. First of all, the study was exclusively carried out on young, healthy females. An extension of our results and conclusions to younger, older or male subjects is not possible without evidence from further research. Secondly, stimuli similar to the ones in our previous studies were used. These stimuli were adapted to our subject group of young females. Although we have shown in earlier publications that these stimuli elicited the desired emotional responses, further evidence is needed to generalize our conclusions to other types of stimuli (e.g., facial expressions of healthy adults and non-facial stimuli) and other emotions (e.g., anger and anxiety). Thirdly, we were not able to incorporate eye tracking in this study. Due to the absence of such a device, we were not able to check whether all volunteers remained focused on the stimulus presentation.

## Conclusion

In this study, we have extended the previously reported relationship between the personality trait HA and the neural activity generated while passively viewing or implicitly processing affective stimuli, to the situation where these stimuli are processed explicitly. The results obtained, pointed in the high HA participants to a higher activity in the visual cortex and facial processing areas and in the prefrontal cortex. The enhanced facial processing boosts the activity in the left LB amygdala, while the increased cognitive control successfully inhibits any increased emotional response in the CM amygdalae in high HA females.

## Consent

Written informed consent was obtained from the participating volunteers for the publication of this report and any accompanying images.

## Abbreviations

ANOVA: Analysis of variance; ACC: Anterior cingulate cortex; CM: Centromedial; DLPFC: Dorsolateral prefrontal cortex; fMRI: Functional magnetic resonance imaging; GLM: General linear model; HA: Harm avoidance; HRF: Hemodynamic response function; LB: Laterobasal; MCC: Middle cingulate cortex; OFC: Orbitofrontal cortex; SF: Superfisial; TCI: Temperament and character inventory; VLPFC: Ventrolateral prefrontal cortex.

## Competing interests

The authors declare that they have no competing interests.

## Authors’ contributions

Design of the study: PV, CB. Acquisition of data: PV, CB. Analysis and interpretation of data: PV, CB. Writing the manuscript: PV. Revising the manuscript critically: CB, RD, RL, JD. All authors read and approved the final manuscript.

## Supplementary Material

Additional file 1 **Significant responses to the positive stimuli.** These results were found after applying a voxel significance threshold *p* < 0.005 and a cluster-extent threshold Ke > 767 voxels. Click here for file

Additional file 2 **Significant responses to the negative stimuli.** These results were found after applying a voxel significance threshold *p* < 0.005 and a cluster-extent threshold Ke > 762 voxels. Click here for file
